# Micro mercury trapped ion clock prototypes with 10$^{-14}$ frequency stability in 1-liter packages

**DOI:** 10.1038/s41598-023-36411-x

**Published:** 2023-06-30

**Authors:** Thai M. Hoang, Sang K. Chung, Thanh Le, Sehyun Park, Sung-Jin Park, J. Gary Eden, Christopher Holland, Hao Wang, Omeed Momeni, Russell Bradley, Scott Crane, John D. Prestage, Nan Yu

**Affiliations:** 1 Jet Propulsion Laboratory, California Institute of Technology, Pasadena, CA 91109 USA; 2 Laboratory for Optical Physics and Engineering, Department of Electrical and Computer Engineering, University of Illinois, Urbana, IL 61801 USA; 3 Applied Sciences Division, SRI International, Menlo Park, CA 94025 USA; 4 Department of Electrical and Computer Engineering, University of California, Davis, CA 95616 USA; 5 Advanced Space PNT, Space Systems Development, United States Naval Research Laboratory, Washington DC, DC 20375 USA; 6 Present Address: Envisioneering, Inc., 5904 Richmond Hwy, Ste. 300, Alexandria, VA 22303 USA

**Keywords:** Physics, Applied physics, Atomic and molecular physics, Electronics, photonics and device physics, Optical physics, Quantum physics, Techniques and instrumentation

## Abstract

Modern communication and navigation systems are increasingly relying on atomic clocks. As timing precision requirements increase, demands for lower SWaP (size, weight, and power) clocks rise. However, it has been challenging to break through the general trade-off trend between the clock stability performance and SWaP. Here we demonstrate micro mercury trapped ion clock (M2TIC) prototypes integrated with novel micro-fabricated technologies to simultaneously achieve high performance and low SWaP. The M2TIC prototypes could reach the $10^{-14}$-stability level in 1 day with a SWaP of 1.1 L, 1.2 kg, and under 6 W of power. This stability level is comparable to the widely used rack-mount Microchip 5071A cesium frequency standard. These standalone prototypes survived regular commercial shipping across the North American continent to a government laboratory, where their performance was independently tested. The M2TIC sets a new reference point for SWaP and performance and opens opportunities for high-performance clocks in terrestrial and space applications.

## Introduction

**Figure 1:**
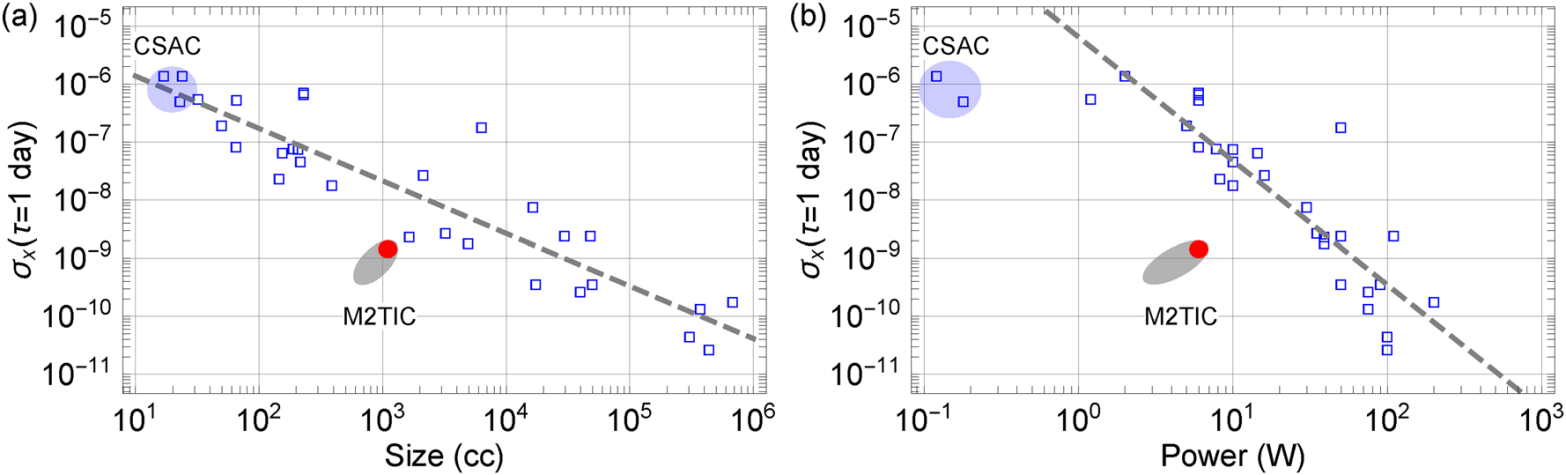
Time deviation after a day vs. size (**a**) and power (**b**) of some of the state-of-the-art compact atomic clocks. The compact atomic clock data (blue markers) are taken from Ref.^1^ and the references within for Microchip CSAC, Teledyne TCSAC, Microchip 5701A, FEI RAFS, Excelitas RAFS, Microchip MHM, and many others. The solid red dot indicates the current M2TIC prototypes, and the gray shaded area indicates further improvements that can be made in the near future.

The quantum technology of the atomic clock has made GPS possible and unambiguously changed our daily lives. Atomic clocks provide precise timing signals for modern communication, positioning, and navigation systems. Future atomic clocks will emphasize not only the performance but also size, weight, and power (SWaP)^1^. However, developing a new atomic clock system is like playing with a seesaw where SWaP and performance sit on the opposite ends of a balance. A higher performance atomic clock usually requires more atoms, better vacuum, higher laser powers, and more complicated system and environment control, leading to a larger size and higher electric power consumption. During the past few decades, there have been a number of different approaches for miniature atomic clocks^1,2^, from vapor-cell-based clocks^3–18^ to laser-cooled and trapped neutral atoms^19–22^ and ions^23–48^. Despite many efforts, few approaches were able to move beyond breadboard demonstrations and made to a standalone clock system. It has proved difficult to miniaturize a practical atomic clock while maintaining high performance. Such a trend is best illustrated in Fig. 1, which shows the time deviation after one day vs. the clock system size for well-known commercial and operational atomic clocks. A typical trade-off trend between the size and power is evident; a higher stability clock requires a correspondingly larger size (and power) to achieve. Recently, we have developed the micro mercury trapped ion clock (M2TIC) technology and constructed three completely packaged prototypes that significantly break away from the general trend as shown in Fig. 1.

**Figure 2:**
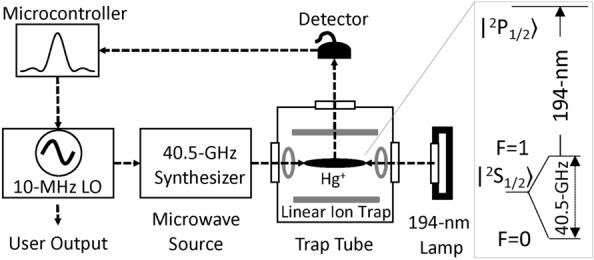
A simplified mercury-ion clock. The $^{199}$Hg$^+$ ions are trapped by a linear RF trap inside a vacuum trap tube. The inset shows relevant energy levels for the trapped ions. The clock transition is the 40.5-GHz microwave transition from the $\mid S_{1/2},F=0 \rangle$ to $\mid S_{1/2},F=1 \rangle$ state. The clock transition is interrogated by a 40.5-GHz microwave pulse, which excites the ions from the lower clock state to the upper clock state. To detect the excited ions, a 194-nm light pulse from a $^{199}$Hg$^+$ lamp optically pumps the ions from the upper clock state to the lower clock state through the $\mid P_{1/2} \rangle$ state. A photomultiplier tube detector collects the fluorescence photons as the ions spontaneously decay from the $\mid P_{1/2} \rangle$ to $\mid S_{1/2} \rangle$ state. The microcontroller uses the ion signal to lock the 10-MHz LO frequency to the atomic clock transition. The dashed arrows show directions between components.

**Figure 3:**
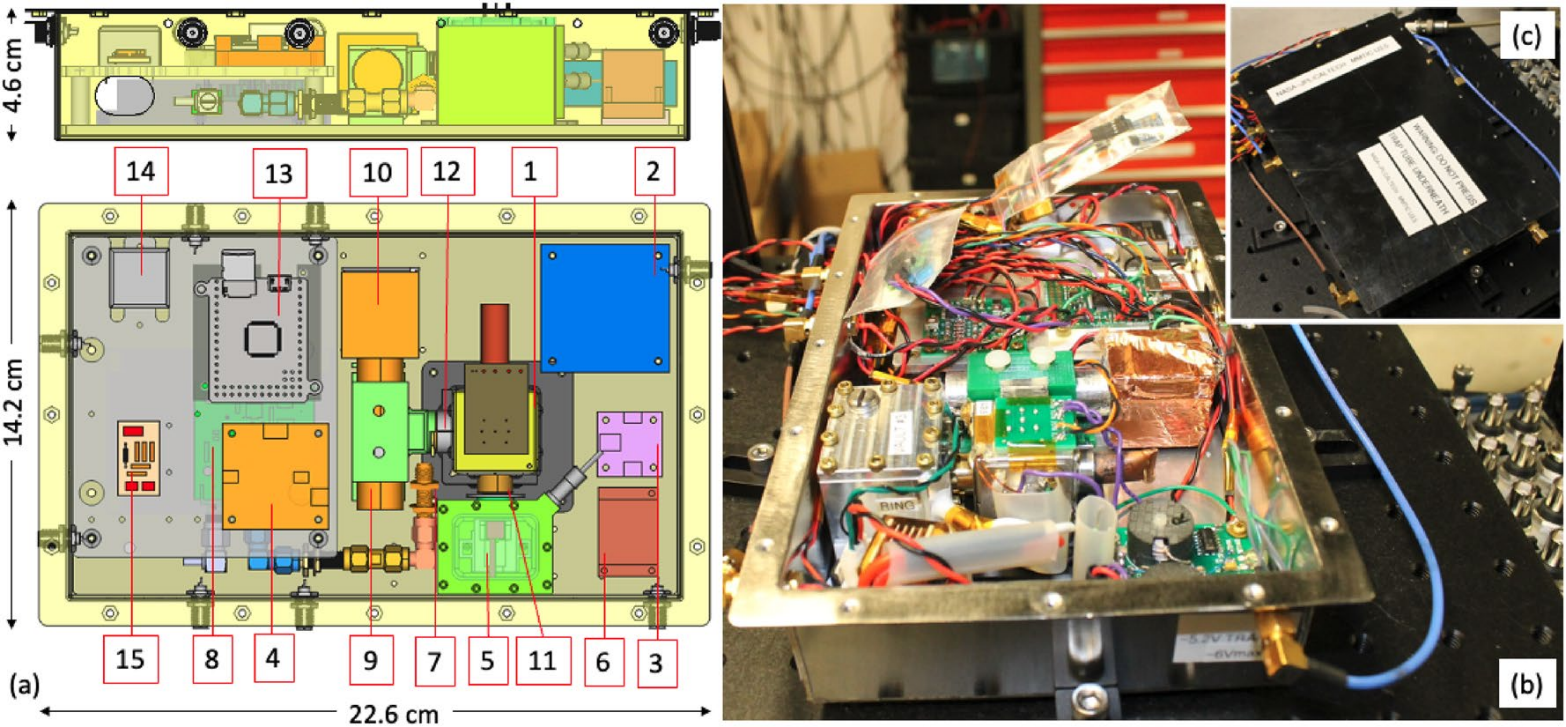
(**a**) Computer-aided design the clock assembly designated as U3.5. The entire package is 14.2 cm$\times$22.6 cm$\times$4.6 cm. The numerical labeling indicates the following: 1, trap tube and inner magnetic shield; 2, trap RF driver; 3, C-field current driver; 4, FEA voltage source; 5, helium vault with a micro mercury lamp inside; 6, HV lamp driver; 7, 40.5 GHz microwave antenna; 8, CMOS Synthesizer (green board under the middle gray plate); 9, PMT UV detector; 10, PMT HV supply; 11, lamp-to-ions optics; 12, ions-to-PMT optics; 13, microcontroller; 14, 10-MHz quartz local oscillator; 15, voltage control board for the 10-MHz oscillator. (**b**) Picture of actual component placements in U3.5. (**c**) The inset shows an enclosed clock package, where the enclosure is the outer magnetic shield.

The M2TIC approach leverages the previous trapped mercury ion clock development efforts^32–36,39–43^. Since the 1990s, the mercury trapped ion approach has been identified as a promising candidate for a compact atomic clock, especially as a space clock. More recently, it was developed into the Deep Space Atomic Clock (DSAC) that was successfully flown in the NASA DSAC mission^42^. In all these mercury ion clocks, conventional components such as thermionic filament electron source, inductive discharge lamps, and frequency multiplier-based microwave synthesizers were used. These components are large and dominate the overall system power consumption. Focusing on these key components, in M2TIC, we developed miniature vacuum trap tubes with field-emitter-arrays (FEA) electron source^44–49^, 194-nm microplasma lamps^45–48,50–54^, and 40.5-GHz CMOS-based microwave synthesizers^55–57^. With all these novel technologies integrated into the M2TIC clock packages and improvements in electronics, we constructed three complete standalone prototypes. These prototypes were carefully characterized and evaluated. They were also sent to the U.S. Naval Research Laboratory (NRL) for independent evaluations. The results show that the M2TIC prototype clocks can average down to the low $10^{-14}$-fractional frequency stability floor, better than that of typical Rb GPS clocks and comparable with the rack-mount 5071A cesium atomic frequency standard widely used for high-performance clock applications. Yet these prototypes have a significantly lower SWaP than other similar performance clocks, with 1.1 L in size, 1.2 kg in mass. The M2TIC prototype consumes less than 6 W of DC electric power.

## Results

### Integrated prototypes

The basic architecture of M2TIC is illustrated in Fig. 2^30–37,39–48^. $^{199}$Hg$^+$ ions are trapped by a small linear quadrupole ion trap (5 mm $\times$ 5mm $\times$ 15 mm) consisting of four rods and two endcaps. An FEA is integrated onto the trap electrodes inside the tube. A 2-MHz trap RF driver generates a 450-V peak-to-peak voltage on the trap rods with opposite phases on adjacent rods. A 17 VDC is applied between the trap rods and the endcaps to enclose the ion trap. The trap driver consumes about 250 mW of DC power. The ion trap is housed inside a 15-cc miniature vacuum trap tube made of titanium walls and sapphire optical windows filled with mercury vapor and helium buffer gas (see “Method”). These particular construction materials can be used to construct a vacuum trap tube without an active ion pump for various atomic species.

**Figure 4:**
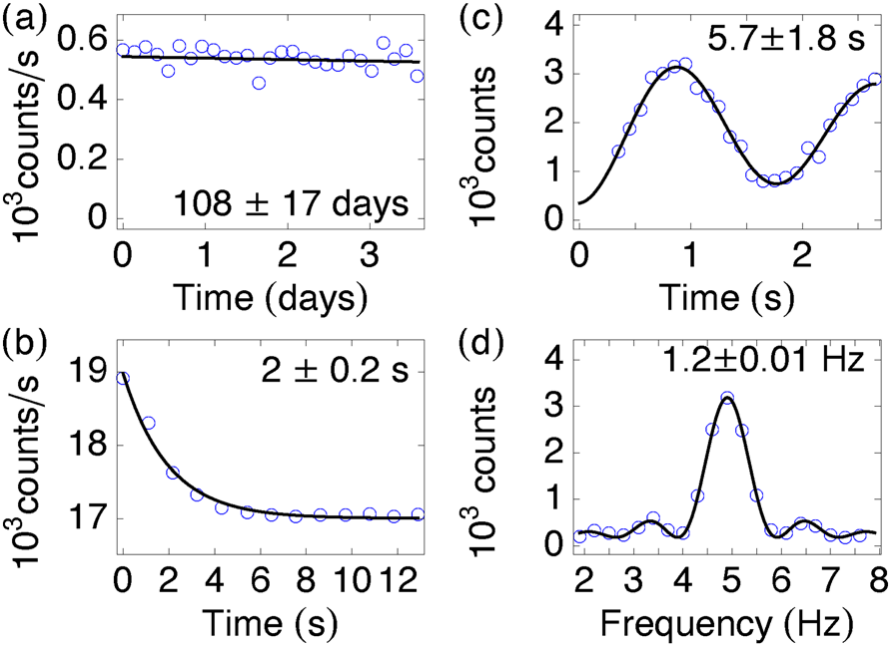
(**a**) The ion lifetime of $108 \pm 17$ days is obtained by fitting the data (open circles) to an exponential function (solid curve). (**b**) The optical pumping time of $2 \pm 0.2$ s is obtained by fitting the data (open circles) to an exponential function (solid curve). (**c**) The Rabi oscillation data (open circles) are plotted after background subtraction. The coherence time of $5.7 \pm 1.8$ s is obtained by fitting the data to a decaying sinusoidal function (solid curve). (**d**) The linewidth data are plotted after background subtraction. The linewidth of $1.2 \pm 0.01$ Hz is obtained by fitting the data (open circles) to a sinc$^2$ function (solid curve).

To initiate the clock operation from cold, the FEA is activated to ionize the mercury vapor to load $^{199}$Hg$^+$ ions into the ion trap. The ion loading process could be completed within minutes; however, the FEA current was typically set at a few $\upmu A$ to fully load ions in 20 min. The FEA consumes about an mW of DC power, whereas a thermionic electron source generally consumes several watts^44,48^.

For the clock operation, the $^{199}$Hg$^+$ ions must be first optically pumped to the lower clock hyperfine state. This is accomplished by using the light emission from a $^{202}$Hg discharge lamp, which has a partial spectral overlap with one of the $^{199}$Hg$^+$ ion clock hyperfine states^30,31,58^. While there exists no low-SWaP 194-nm laser that fits this purpose, the plasma discharge lamp method offers simplicity and robustness as a light source. It does not need wavelength locking, optical isolation, modulation, or optical shutter typically required for a laser source. To minimize the size and power for the light source, we moved away from the conventional inductive discharge lamp and instead adapted the microcavity plasma discharge lamp technology already used in the lighting and sanitization industries^50–53^. We have developed a small, efficient 194-nm mercury microplasma lamp of dielectric barrier discharge configuration. A microplasma lamp was fabricated from a fused silica cell, which consisted of an array of microcavities (0.6–1.0 mm in diameter). The lamp was injected with a few mg of $^{202}$Hg and was filled with a few hundred torrs (over 20,000 Pa) of helium^45–48,54^. The high efficiency comes from the smaller size, the field-enhancing microcavities, and the direct energy transfer process from the helium molecular dimers in the discharge. This approach significantly reduced the total power consumption of the 194-nm microplasma lamp to about 300 mW. For comparison a conventional inductive mercury discharge lamp consumes over 10 watts of power^32–37,39–43^.

The Hg$^+$ clock transition is the 40.5-GHz transition from the $\mid S_{1/2}, F=0 \rangle$ to $\mid S_{1/2}, F=1 \rangle$ states. This high microwave frequency provides a high quality factor and low magnetic sensitivity desired for a high-performance clock. At the same time, it makes the clock frequency source generation more challenging. A synthesizer is needed to generate the 40.5 GHz signal that is phase locked to a quartz local oscillator (LO). Two of the prototypes used a commercial-of-the-shelf (COTS) synthesizer that consumes over 2.5 W of DC power. This conventional synthesizer uses frequency multipliers that are power hungry. In the same clock development program, we developed a CMOS-based synthesizer scheme that bypasses part of the multiplication process by the sub-sampling phase-locked loop (SSPLL) technique^55–57^. The SSPLL chip was fabricated using a 65-nm CMOS process. The 40.5-GHz CMOS frequency synthesizer consists of a 900 MHz phase-locked loop (PLL) cascaded with a 40.5 GHz SSPLL to achieve the necessary large multiplication factor of 2025 from the designed 20 MHz input frequency. The first stage of the low-frequency PLL consumes only 1 mW while the entire multiplier part from the 20 MHz LO input to the 40.5 GHz clock signal consumes about 40 mW of power. At the current stage, some additional discrete parts were used with the CMOS synthesizer chip to work in the current clock system. This CMOS system was successfully used in one of the prototypes, demonstrating the future power reduction for M2TIC clocks when it is fully implemented.

We also implemented a low-power RF trap drive, DC supplies to trap bias, FEA and C-field coil, and a microcontroller for the prototype clocks. Integrating and packaging all these technologies and components into a small standalone unit is another challenge. Attentions were paid to the placements of components, mechanical consideration, thermal management, wire routing, and RF interferences. Overall, we have built three standalone prototypes. They were packaged in a box of the same design and dimension made of mu-metal, which also acted as a second layer of the magnetic shield. The first inner layer of the shield was around the trap tube itself^48^. We referred to these units as U3.2, U3.4, and U3.5. In U3.2 and U3.4, we used the COTS 24-GHz synthesizer system with a doubler to 40.5 GHz. In U3.5, a low-power CMOS synthesizer chip was placed in a circuit board with supporting electronic components. The supporting components were housed in a separate small box. The lamp in the U3.5 was housed in a vault to prolong the lamp lifetime (see “Method). Figure 3 shows the computer-aided design and actual package clock unit for U3.5. It is clear that the mu-metal clock box has more room to spare, and the clock package can still be smaller.

**Figure 5:**
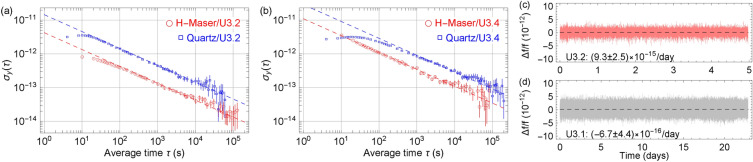
Allan deviation measurements of the prototype U3.2 (**a**) and U3.4 (**b**) using a hydrogen maser LO (red circles) and quartz LO (blue squares). Data are fitted to the $\tau ^{-1/2}$-function (dashed lines). The errorbars represent the estimated uncertainty of the Allan deviation, which is calculated as $\sigma _y(\tau )/\sqrt{n}$. Here *n* is the number of measurements. The raw frequency measurements for U3.2 and U3.1 are plotted in (**c**) and (**d**). The numbers represent the frequency drifts and fitting uncertainties with a linear function.

### Initial characterizations

After completing a prototype clock, we first measured the ion storage lifetime. Here we monitored the ion signals with and without microwave excitation over time. Without the microwave, the ions were optically pumped by the 194-nm light to the lower clock state (dark state), and the ion signal was suppressed close to the background level. Figure 4a shows the ion signal after the background subtraction. The ion lifetime in unit U3.5 was over 100 days, indicating the ability to achieve an excellent vacuum condition without any active pumping. While we do not have a direct way to measure the vacuum condition in the sealed trap tube, the measured lifetime is far longer than those of ions in an actively pumped system. The long ion lifetime helps the clock operation and is critical to achieving long-term stability by reducing the ion number-dependent second-order Doppler shift.

We also characterized the optical pumping and Rabi rates as these measurements help determine an optimal timing sequence for the clock operation. Figure 4b shows an example of optical pumping in U3.5. Here the ions were initially blasted with a high microwave power pulse. The ion population in the upper and lower clock states were roughly equalized. After the microwave pulse ended, the ion signal decayed exponentially as the 194-nm microplasma lamp optically pumped the ions into the dark state. The optical pumping was about 2 s, determined from the exponential fitting. Figure 4c shows a Rabi oscillation measurement in U3.5 using a CMOS synthesizer. The Rabi measurement showed the ion signals after different periods of microwave excitation. The Rabi oscillation yielded a $\pi$-pulse time of about 0.8 s. The coherence time of the clock transition was about 5.7 s, obtained from the decaying sinusoidal fit. This coherence time was much longer than needed as the microwave interrogation was about 1 s. Figure 4d shows a typical microwave spectrum of the ions. The microwave linewidth of 1.2 Hz was obtained by fitting the data to a sinc$^2$ function. This Rabi linewidth was close to the expected Fourier-transform limit of a 0.8-s pulse. The signal-to-noise ratio (SNR) at the resonance peak was about 12, and we generally aimed at an SNR above 7. The ion detection noise was largely limited by the shot noise of the scattered background lamp light.

### Stability measurements

**Figure 6:**
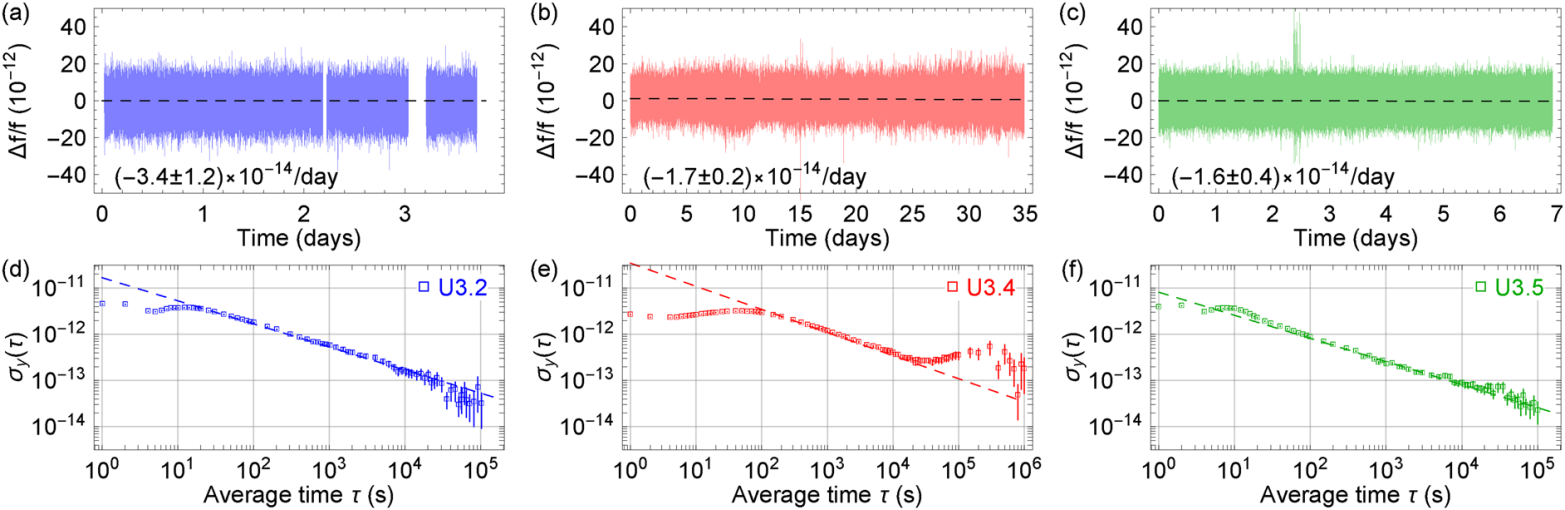
The raw frequency measurements for U3.2, U3.4, and U3.5 are plotted in (**a**)–(**c**). The gaps in the raw data occurred when the clocks were not locked. The numbers represent the frequency drifts and fitting uncertainties with a linear function. Since there are over 3 million raw data points for (**b**), the plot only displays one for every ten data points. The Allan deviations for U3.2, U3.4, and U3.5 are plotted in (**d**)–(**f**). The Allan deviation for each clock is calculated by combining the separate locked segments into a single continuous sequence. The Allan deviation data are fitted to the $\tau ^{-1/2}$-function (dashed lines). The errorbars represent the estimated uncertainty of the Allan deviation, which is calculated as $\sigma _y(\tau )/\sqrt{n}$. Here *n* is the number of measurements.

Based on similar parameters above, we normally operated with a timing sequence of 1 s of microwave interrogation, followed by 2–3 s of optical pumping/detection, and 0.3 s of FEA for the ion loss compensation between interrogation cycles. We first characterized the intrinsic frequency stability of the trapped ion reference using a hydrogen maser (HM) LO. HM has the short-term stability below $10^{-13} \tau ^{-1/2}$ and a stability floor at the level of $10^{-15}$. Figure 5a and b show that both U3.2 and U3.4 can reach a noise floor of $3\times 10^{-14}$ with a short-term stability of $5\times 10^{-12} \tau ^{-1/2}$ and $1\times 10^{-11} \tau ^{-1/2}$, respectively. When an onboard quartz LO is used in a closed loop of an actual clock mode, the frequency stability of both U3.2 and U3.4 reaches below the $5\times 10^{-14}$-level with a short-term stability of $1.5\times 10^{-11} \tau ^{-1/2}$ and $3\times 10^{-11} \tau ^{-1/2}$ respectively. These short-term stabilities of the prototype clocks were limited by that of the quartz oscillators used. The oscillator is a commercial COTS (but otherwise state-of-the-art) lower-power high-stability type. It still consumes over 2 W of DC power. Together with the COTS synthesizers, they dominate the overall clock prototype power consumption of the 6 W demonstrated.

Fitting the raw frequency data of U3.4 yields a frequency drift below $10^{-14}$/day, as shown in Fig. 5c. For reference, a high-performance space-qualified rubidium atomic frequency standard (RAFS) with a SWaP of 4 L, 6 kg, and 39 W typically has a drift of $5\times 10^{-14}$/day^1^, which is 5 times larger. In a separate breadboard clock setup of similar M2TIC components designated as U3.1, the frequency drift is about $7\times 10^{-16}$/day, as shown in Fig. 5d. The frequency drift of U3.1 is 70 times lower compared to the high-performance RAFS. We should note that there is no fundamental difference between the breadboard setup and 1-liter prototypes as they all used similar vacuum trap tubes, optics, and electronics^47^. This suggests the frequency drift of the M2TIC prototypes can be further improved.

We note that there are no initial frequency stability measurement data presented here for U3.5 as it was not performed at JPL before shipping it to the Naval Research Laboratory (NRL) for government verification tests (see below).

### Stability measurements at NRL

All three prototypes were shipped to NRL to independently validate the clock performance. The shipments were carried out by common commercial carriers. In some shipments, the tilt and shock indicators were triggered. Nevertheless, all three prototypes arrived intact and operational after some operational condition changes and software updates. In fact, the software on U3.5 was not tested at JPL before being installed at NRL.

Figure 6 shows a set of raw frequency data and the Allan deviations for the prototype clocks measured at NRL. Fitting the raw frequency data to a linear function indicates a frequency drift as low as $1.6\times 10^{-14}$/day, as shown in Fig. 6a–c. Figure 6d shows that U3.2 can reach a fractional frequency stability of $3\times 10^{-14}$ after a day of averaging with a short-term stability of about $2\times 10^{-11} \tau ^{-1/2}$. The Allan deviation measurement of U3.4 is shown in Fig. 6e with a short-term stability of $3\times 10^{-11} \tau ^{-1/2}$. The Allan deviation of U3.4 has a bump around an averaging time of $10^5$ s; nevertheless, the Allan deviation trends toward the $10^{-13}$ level and beyond at longer averaging times. As U3.4 stayed locked exceptionally well right after the first try, we decided to leave U3.4 running without an optimization attempt for a remarkable period of nearly 35 days continuously. Out of three prototypes, U3.5 has the best stability performance with a short-term stability of $10^{-11} \tau ^{-1/2}$. U3.5 can reach a fractional frequency stability of $2\times 10^{-14}$ after a day of averaging as shown in Fig. 6f.

We also quantified frequency retraces of the prototype clock. Here the output clock signals were observed over a brief outage or discontinuity in service, varying between minutes to 60 h. The average frequency was calculated over a 24-hour period before and after the break. The retrace value is the difference between these two estimations with an uncertainty equal to the root mean square (RMS) of each frequency estimate’s individual error. This method was called the adjacent point differential stencil [DS] method at NRL. U3.2, U3.4, and U3.5 had retraces of $-\,\,6.3\times 10^{-12}$, $-\,\,2.5\times 10^{-12}$, and $4.4\times 10^{-13}$, respectively. Here we report the upper bound of the frequency retraces for each prototype clock. They are consistent with what we previously reported retrace of $1\times 10^{-12}$ in the similar trap tubes^47,48^.

**Table 1:** SWaP and performance of the prototype clocks.

Prototype	U3.2	U3.4	U3.5
Size	1.1 L	1.1 L	1.1 L$^\dag$
Weight	1.2 kg	1.2 kg	1.2 kg$^\dag$
Power	4.6 W	4.6 W	5.9 W
$\sigma _y(\tau )$/HM at JPL	$5\times 10^{-12} \tau ^{-1/2}$	$10^{-11} \tau ^{-1/2}$	
$\sigma _y(\tau )$ at JPL	$1.5\times 10^{-11} \tau ^{-1/2}$	$3\times 10^{-11} \tau ^{-1/2}$	
$\sigma _y$ floor at JPL	$2\times 10^{-14}$	$3\times 10^{-14}$	
$\sigma _y(\tau )$ at NRL	$2\times 10^{-11} \tau ^{-1/2}$	$3\times 10^{-11} \tau ^{-1/2}$	$10^{-11} \tau ^{-1/2}$
$\sigma _y$ floor at NRL	$3\times 10^{-14}$	$<2\times 10^{-13}$	$2\times 10^{-14}$
Drift (day)	$-3.4\times 10^{-14}$	$9.3\times 10^{-15}$	$-1.6\times 10^{-14}$
Retrace (±)	$-6.3\times 10^{-12}$	-$2.5\times 10^{-12}$	$4.4\times 10^{-13}$

$\dag$ U3.5 size and weight do not include the external 20-MHz frequency generator.

## Discussion

The performance and SWaP of the prototype clocks are summarized in Table 1. Overall, the M2TIC prototype demonstration shows a promising path for a new generation of low-SWaP, high-performance clocks. In maintaining a certain level of clock performance, we have to focus on both the short-term and long-term stabilities in the design of the micro clock concept. The short-term stability of the ion reference is determined by the quality factor of the transition and the ion signal-to-noise ratio as characterized by the Allan deviation as,

$$\begin{aligned} \sigma _y(\tau ) =\frac{1}{\pi \times Q \times \mathrm {SNR}} \sqrt{\frac{t_c}{\tau }}. \end{aligned}$$

Here the quality factor $Q=f_0/\Delta f$ with $\Delta f$ being the full width at half maximum (FWHM) of the atomic transition. The $t_c$ is the clock cycle time, and $\tau$ is the average time. Since the COTS quartz LO used can maintain the stability of $3\times 10^{-12}$ up to a few tens of seconds, we typically operate with $\Delta f$ around 1 Hz using an interrogation of 1 s. While a narrower $\Delta f$ will improve the short-term stability, the LO may not stay locked reliably. The SNR is determined partly by the number of ions in the trap, which is, in turn, determined by the trap size and operation parameter. The trap design kept the size to a minimal while its RF driver parameters are optimized to still maintain sufficient ions. Usually, an SNR above 7 will keep the prototype locked robustly. Special attention was also paid to the optical setup for dealing with the incoherent and spatially extended lamp light source to maximize the ion signal while keeping the background light low.

The two dominating factors affecting the long-term stability and the drift are the second-order Zeeman shift and the second-order Doppler shift due to the ion micromotion. The second-order Zeeman shift is common to all atomic clocks. Hg$^+$ has the advantage of being the least magnetic field-sensitive microwave clock species. The key to achieving low field sensitivity is to operate with the lowest possible magnetic field while implementing magnetic shielding. Currently, at least a 10 $\upmu$T bias field has to be used, limited by the residual magnetic field gradient in the trap region. We applied 20 $\upmu$T to provide an ample buffer for the prototypes in case of unexpected changes due to shipping. With the two layers of the magnetic shield, a low $10^{-14}$ stability floor was achieved in a typical laboratory environment. Additional layers of the magnetic shield will reduce the magnetic field sensitivity accordingly, albeit at the expense of mass and volume. The second leading source of frequency instability comes from ion number fluctuations. The average second-order Doppler shift depends on the ion cloud size due to the inherent micromotion associated with the ion trapping by the RF field. Maintaining a steady ion number in the trap is the key to long-term stability. We estimate that a $1\times 10^{-14}$ stability level requires stabilizing the ion number within 2%. In the current trap tubes, trapped ions can have a long lifetime of over a hundred days. The ion number is stable with a negligible ion loss per day. Since the FEA power consumption is low, we can briefly reload ions between clock cycles to compensate for the ion loss and keep the ion number steady over extended periods of operation, as demonstrated on 35 days of continuous operation with U3.4 at NRL.

While the current M2TIC prototypes have made a breakthrough in a typical SWaP and performance trade-off, there are still significant improvements to be gained. More careful layouts and advanced electronics fabrications can reduce the overall size by $50\%$, well under 1 l in the actual volume of the unit. The power consumption can also be further reduced. The two commercial COTS components, the synthesizer and oscillator, alone consume more than $60\%$ of the total DC power consumption of the current prototypes. The whole CMOS-based microwave synthesizer would consume less than 500 mW with proper implementation. There also have been efforts to develop ultra-low power oscillators with the goals of less than 100 mW power consumption. At the same time, the M2TIC has the potential to reach below a $1\times 10^{-14}$ long-term stability, competing with some of the best operational clocks, as demonstrated in the breadboard system^47^. It is reasonable to expect that the M2TIC SWaP can be in a 3 W and 700 mL range, as indicated in the shaded area in Fig. 1.

In summary, three packaged micro mercury ion clocks prototypes were successfully developed and demonstrated. The prototype clocks survived real-world testing with commercial shipping services. The prototype clocks have a short-term stability of about $10^{-11} \tau ^{-1/2}$ and can average down to the $10^{-14}$-stability level in a day. The corresponding frequency drift is less than $10^{-14}$/day and could be improved to below $10^{-15}$/day. This stability performance makes M2TIC comparable with the state-of-the-art atomic clocks of much larger SWaP. With utilization and integration of novel microfabricated technology components, the M2TIC approach has broken through the general trade-off trend of SWaP vs. stability performance of the commercial and operational atomic clocks. The high-performance and low-SWaP clocks will make the M2TIC more ubiquitous in future communication, navigation, and arrayed sensing, including deep space applications.

## Method

### Trap tube fabrication

The trap vacuum tube components were welded together with electron-beam welding since miniaturization cannot be achieved with nuts and bolts. A miniature trap tube allows surrounding components to be in close proximity to the trapped ions and usually reduces the overall power consumption. For instance, a small ion trap will consume less power than that of a large ion trap to produce the same trapping potential. The trap tube was then baked to 400 $^\circ$C to achieve a base pressure of nano-torr ($10^{-7}$ Pa) and a passive non-evaporative getter (NEG) inside was activated at the same time. The trap tube was backfilled with about 3 micro-torr ($4\times 10^{-4}$ Pa) of helium as the buffer gas cooling for the trapped ions. The pressure was measured with an ion gauge without without a gas correction factor applied. The NEG pump can maintain the vacuum integrity in the trap tube for many years with zero power consumption. After the preparation process, the trap tube was pinched off and completed separated from the pumping station.

### Vaulted lamp implementation

Currently, the helium permeation loss through the lamp fused silica cell is the limiting factor for the lamp continuous operating lifetime. To mitigate this limitation and prolong the lamp lifetime, we investigated the effectiveness of the method of enclosing the lamp in a helium-filled vault. We constructed vaults made of aluminum with a fused silica window. Filling the aluminum vault with helium to about the same pressure as the lamp cell can help maintain the helium level inside the lamp. A vaulted lamp was installed in U3.5 and showed a promising performance of longer lamp operating time. Nevertheless, the fused silica window still needs to be replaced with a sapphire one, for example, to completely eliminate the helium permeation issue. Currently, the vaulted microplasma lamp system consumes about 700 mW of power compared to a 300 mW power consumption of a typical bare lamp. We attribute this behavior to the capacitance change due to the aluminum vault. Modifying the lamp driver circuit specifically for the helium lamp vault system or non-metal vault construction can limit the increase in the power consumption.

### Clock operation

The clock was operated in the Rabi mode, where pulses of 40.5-GHz microwave radiation and 194-nm light were alternatively switched on and off. We normally operated the clock with a timing sequence of 1 s of microwave interrogation, followed by 2–3 s of optical pumping/detection, and 0.3 s of FEA for the ion loss compensation between interrogation cycles. Here the fluorescence from the optical pump process is also used as the state detection signal. For the detection, a photomultiplier tube (PMT) collects the emitted photons as the ions decay from the $\mid P_{1/2}\rangle$ to $\mid S_{1/2} \rangle$ state during the optical pumping period. A microcontroller applies a tuning voltage to the 10-MHz oscillator based on the ion signal detected by the PMT to close the clock loop. In practice, the microwave frequency is switched alternatively between the high and low frequency sides of the microwave resonance line. After each measurement, a feedback voltage is calculated proportionally to the difference between the two most recent PMT counts (times with a gain factor) and added to the tuning voltage.

### Stability measurements

The stability of the prototype clocks were characterized against a HM reference. To measure the intrinsic frequency stability of the trapped ions, the 40.5-GHz clock transition frequency was tracked in the open loop mode, where the microwave system is referenced to the HM reference. To evaluate the stability of a prototype clock in the closed loop mode, the 10-MHz frequency output was measured using a commercial Phase Noise and Allan Deviation Test Set. At NRL, the 10-MHz frequency output was measured using Code 8150’s in-house Multiple Measurement System (MMS) with reference to a hydrogen maser.

## Data Availability

Raw data for all figures are available from the corresponding author upon request.
